# The Ubiquitin Script: writing protein fates in chains

**DOI:** 10.1042/EBC20253031

**Published:** 2026-04-22

**Authors:** Devanshi Gupta, Subbareddy Maddika

**Affiliations:** 1 Laboratory of Cell Death & Cell Survival, Centre for DNA Fingerprinting and Diagnostics (CDFD), Uppal, Hyderabad, 500039, India; 2 Graduate studies, Regional Centre for Biotechnology, Faridabad, 121001, India

**Keywords:** protein degradation, ubiquitin, ubiquitin chain, ubiquitin linkage

## Abstract

Ubiquitination is a fundamental post-translational modification that orchestrates a wide range of cellular processes. This modification is executed through a cascade of enzymatic steps involving E1 activating enzymes, E2 conjugating enzymes, and E3 ligases. Among these, E2 enzymes and specific E3 ligases primarily dictate the type of ubiquitin linkage formed. Ubiquitination system can form chains of ubiquitin on any of its seven lysine residues or its N-terminal methionine, each generating a distinct three-dimensional topology. These structurally diverse polyubiquitin chains are selectively recognized by ubiquitin receptors, influencing substrate stability, localization, and interactions. These topologically diverse polyubiquitin chains function as discrete molecular signals, each with distinct physiological outcomes. This review focuses on key developments in our understanding of how specific ubiquitin linkage types participate in various cellular pathways and their implications on the fate and function of the protein.

## Introduction

**Figure 1:**
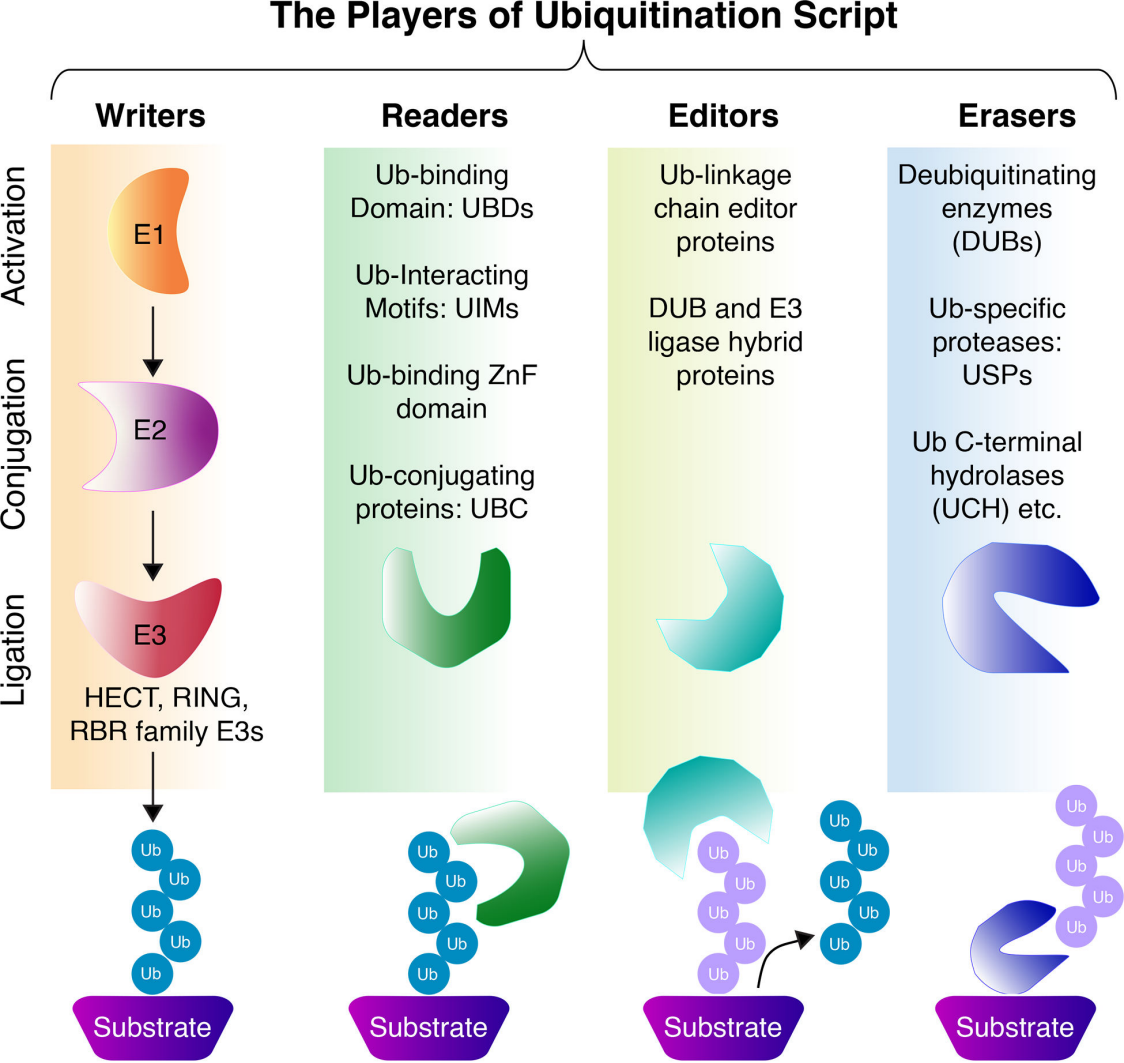
Players of the Ubiquitin Script.

Protein ubiquitination is a highly conserved post-translational modification in eukaryotic cells that involves the covalent attachment of the 76-amino acid ubiquitin (Ub) to target proteins. This process is orchestrated by a sequential enzymatic cascade consisting of a ubiquitin-activating enzyme (E1), ubiquitin-conjugating enzymes (E2s), and ubiquitin ligases (E3s), which collectively determine the substrate specificity and type of ubiquitin modification [1]. The *Ubiquitin Script* is dynamically written, read, edited, and erased by distinct components of the ubiquitination machinery (Figure 1). The functional diversity of polyubiquitin chains arises from their specific synthesis by E2 and E3 enzymes, recognition by ubiquitin readers, and removal by de-ubiquitinating enzymes [2,3]. Ubiquitination, predominantly occurs on lysine residues of substrate proteins, which were long considered the sole and canonical ubiquitination sites. However, recent studies have expanded this view, revealing that cysteine [4,5], serine [6,7], and threonine residues [8,9] as well as the free amino group at the protein N-terminus [10–12] can also serve as ubiquitination sites, forming thioester, hydroxyester, and peptide linkages, respectively [13].

The process of ubiquitination involving the writers, readers, editors, and erasers to mediate substrate ubiquitination has been shown.

**Figure 2:**
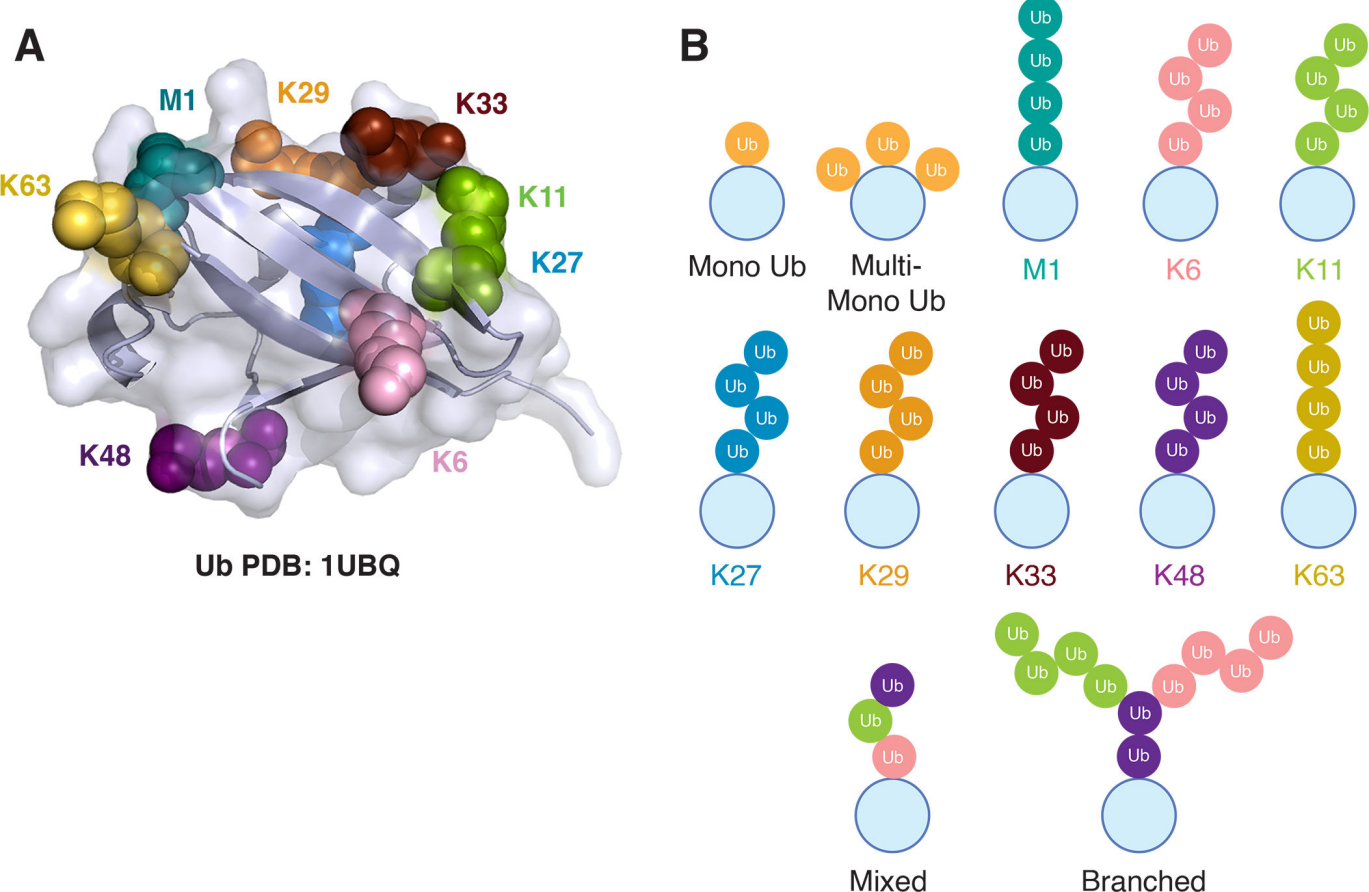
Ubiquitin and types of linkages.

Substrate proteins can be modified with a single ubiquitin (mono-ubiquitination), multiple single ubiquitin (multi-mono-ubiquitination), or by assembling short or extended polyubiquitin chains. Chain elongation occurs when the N-terminal methionine (M1) or one of the seven lysine residues (K6, K11, K27, K29, K33, K48, or K63) of an already conjugated ubiquitin serves as the linkage site for an additional ubiquitin (Figure 2A and B). When the same linkage type is repeated, a homogeneous (homotypic) chain forms, whereas modification via different ubiquitin linkages results in heterogeneous (heterotypic or branched) chains. These structural variations significantly influence the fate of ubiquitinated substrates [14,15]. Based on structural and mechanistic differences, E3 ubiquitin ligases are broadly classified into three major types: RING (Really Interesting New Gene), HECT (Homologous to E6-AP Carboxyl Terminus), and RBR (RING-between-RING). Each class is defined by conserved structural motifs and distinct catalytic mechanisms that govern how ubiquitin is transferred to substrate proteins [16]. E3 ubiquitin ligases mediate the transfer of activated ubiquitin from E2 to specific lysine residues on substrate proteins. RING ligases promote ubiquitination indirectly by facilitating ubiquitin discharge from E2s, thus relying on E2 specificity to determine whether mono- or polyubiquitination occurs and which linkage type is formed [16]. For example, RING E3 BRCA1 can mediate ubiquitination through K6-, K48-, or K63-linked chains depending on its associated E2 [17]. In contrast, linkage-specific HECT and RBR ligases form a thioester intermediate with ubiquitin before transferring it to the substrate, enabling them to directly dictate the ubiquitination outcome [18,19].

**(A**) Structure of ubiquitin (PDB: 1UBQ) highlighting the seven lysines and methionine group. (**B**) Pictorial representation of the kinds of ubiquitin chain types.

The first evidence that all seven lysine residues of ubiquitin can form linkages came from a mass spectrometry–based analysis of ubiquitinated proteins in yeast [20]. This study provided an initial quantitative estimate of linkage abundance, identifying K48 as highly abundant, K63 and K11 as moderate, and K6, K27, K29, and K33 as less prevalent. Subsequently, proteomic profiling by several independent studies for the abundance of polyubiquitin linkages in mammalian cells revealed that K48 and K63 constitute a major fraction of the ubiquitinome, followed by the atypical linkages like K29, K11, M1, K6, K27, and K33 chains [21–23].

**Figure 3:**
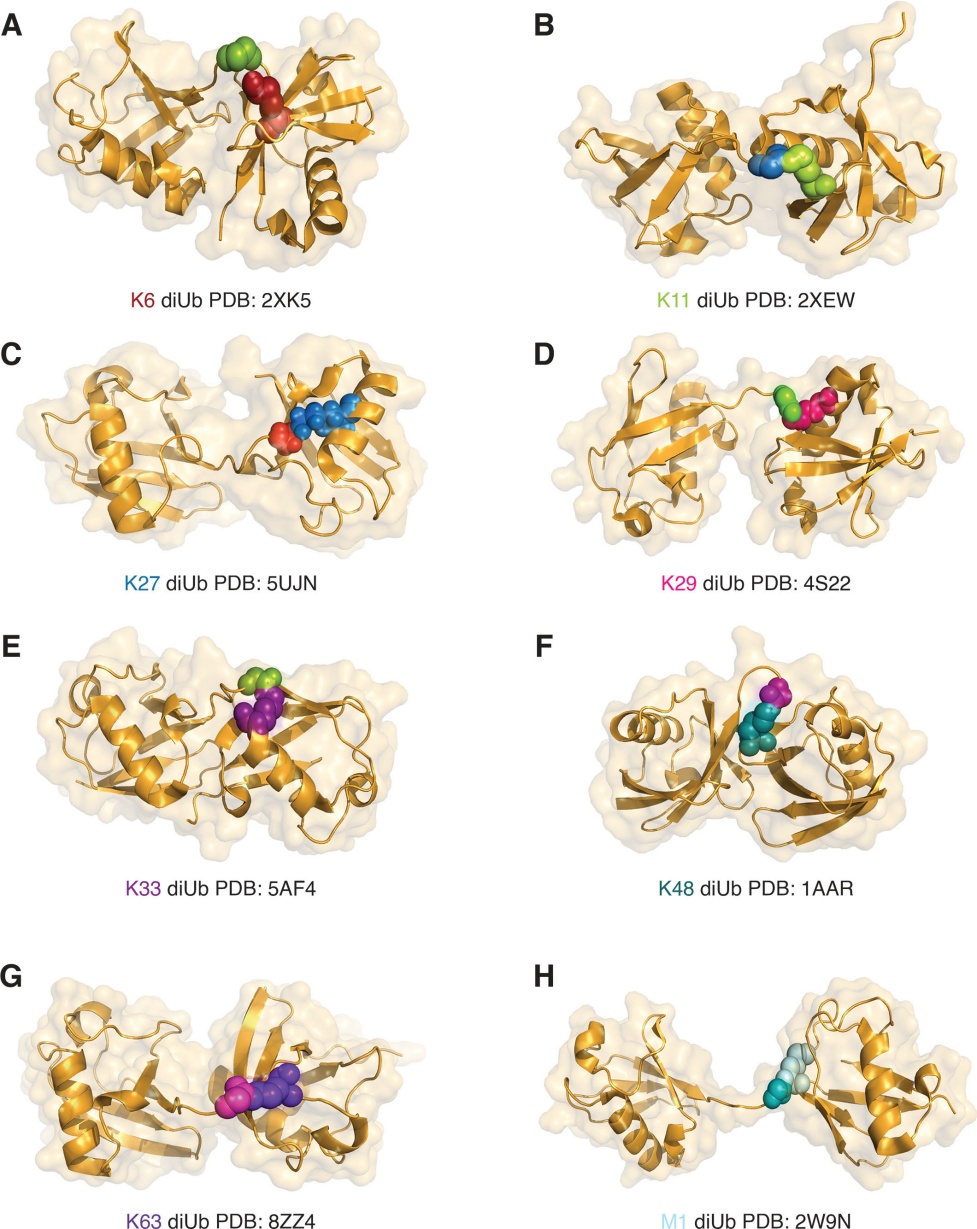
Structures of Ub linkages.

A major challenge in ubiquitin biology lies in deciphering how distinct polyubiquitin linkages are specifically read by ubiquitin readers, as this recognition governs the functional outcome. Each linkage type adopts a unique three-dimensional conformation (Figure 3), enabling selective interaction with downstream machinery that directs processes such as proteasomal degradation, signal transduction, DNA repair, or autophagy [24]. One of the major ubiquitin readers is VCP/p97 (Valosin-containing protein), a type II AAA+ATPase that functions as a central regulator of protein quality control and degradation [25]. This hexameric enzyme recognizes ubiquitinated substrates and enables their recycling or proteasomal degradation, thus acting as a ‘segregase’ [26]. Often, VCP-mediated unfolding becomes a prerequisite for degradation by the proteasome, thus highlighting its functional importance in cellular milieu [27,28]. Although polyubiquitin chains are often described as discrete molecular signals with distinct outcomes, their functional specificity is not absolute. Many ubiquitin readers show limited discrimination between chain types, and different linkages can converge on similar phenotypes, especially when chains cross-talk with one another or with other PTMs. Additionally, the biological context, including the abundance and availability of specific readers, can strongly influence the fate of a given chain type. In several cases, insufficient experimental evidence further blurs these distinctions, contributing to ambiguity and seemingly contradictory observations.

**(A-H**) Structural conformations of unbound di-ubiquitin linked chain types with their respective PDB IDs are shown.

In this review, we aim to provide a comprehensive overview of ubiquitin chain topology, addressing recent advances that remain underrepresented in existing literature. We bridge the knowledge gap concerning the functional diversity of ubiquitin linkages by discussing the players that mediate their formation and removal, the biochemical and structural mechanisms underlying their recognition, the methodologies employed to identify the linkages and the varied cellular processes they regulate. By integrating these perspectives, this review offers a unified understanding of how ubiquitin chain topology dictates cellular fate and highlights the emerging complexity of ubiquitin signaling network.

### Mono-ubiquitination

The initial evidence of mono-ubiquitin as a post-translation modification emerged with the finding that histones H2A and H2B are mono-ubiquitinated in transcriptionally active chromatin [29]. Notably, mono-ubiquitinated H2B hinders the refolding of nucleosome and promotes multiple rounds of transcription [30]. Additionally, mono-ubiquitination also promotes the ligand-induced internalization from plasma membrane of a GPCR [31], a maltose transporter [32], a galactose transporter Gal2p [33], and a plasma membrane protein Pma1p [34] for endocytosis in yeast. In another example, mono-ubiquitination was shown to activate FANCD2 in response to DNA damage and promote its colocalization with BRCA1 at repair foci during Fanconi Anemia [35].

Contrary to the belief that polyubiquitin signal is essential for proteolysis, there are studies where mono-ubiquitination has led to proteolytic degradation. For example, mono-ubiquitination of β-galactosidase and α-globulin acts as a degradation signal and affects their half-lives [36,37]. Mono-ubiquitination can serve as an initiation site for subsequent extension to poly-ubiquitination, thus acting as a signal for unassembled or orphan protein subunits—especially during stress, aging, or aneuploidy—to be targeted for degradation. E4 mega complex UBR4-KCMF1 ubiquitin ligase complex recognizes such orphan proteins and their monoubiquitin to build a polyubiquitin chain for proteasomal degradation [38].

### K6-linked chains

K6-linked ubiquitin chain was first reported as a form of autoubiquitination by the heterologous BRCA1/BARD1 RING ubiquitin ligase [39], and was later demonstrated to be crucial for BRCA1 cellular activity during S-phase and in response to replication stress and DNA damage [40]. The K6-linked ubiquitin chains are recognized by UBXN1 in the BRCA-mediated DNA repair pathway [41,42]. Additionally, BRCA1/BARD1 mediates K6-linked ubiquitination on Nucleophosmin/B23 (NPM) [43], on RNA polymerase subunit RPB8 to regulate cell survival [44], and on CtIP to regulate G2/M checkpoint control [45] after DNA damage. The steady-state levels of all of these substrates were unchanged upon BRCA1/BARD1 ubiquitination, suggesting that K6-linked ubiquitination does not promote degradation. Apart from this, Mahogunin RING finger-1 (MGRN1) also attaches a K6-linked ubiquitin chain on α-tubulin to regulate its polymerization and spindle orientation during mitosis [46]. The K6-linked ubiquitin chains were also shown to be assembled by an RBR E3 ligase Parkin on Mitochondria Outer Membrane (MOM) proteins [47,48]. These linkages are hydrolyzed by the mitochondrial DUB USP30 [49], and USP8 [50] specific for K6 and play an important role in mitophagy and mitochondria quality control. A proteomic screen using affimers for K6 linkage revealed a HECT E3 ligase HUWE1 as the major E3 ligase for K6-linked ubiquitin chains and showed mitofusin-2 as its K6-linked substrate [51].

Even though K6-linked ubiquitin chains do not directly promote proteasomal degradation, they accumulate rapidly after proteasomal and VCP ATPase inhibition [21,52], suggesting that prolonged accumulation of K6-linked ubiquitin chains may eventually lead to degradation. This could occur if these chains either associate with certain cofactors or get further modified with other ubiquitin chains responsible for degradation. For example, during translation, reactive aldehydes induce RNA–protein cross-links (RPCs) that stall ribosomes and block translation. The RBR E3 ligase RNF14 catalyzes K6- and K48-linked ubiquitination on the RPCs at these stalled ribosome–mRNA complexes [53,54]. These ubiquitinated products are then resolved by VCP and degraded by proteasome to restore translation [53]. Moreover, during viral infection, interferons up-regulate acetyltransferase HAT1, which acetylates Viperin and promotes its K6-linked ubiquitination by E3/E4 hybrid ligase UBE4A, resulting in Viperin degradation and poor antiviral response [55]. Additionally, pathogenic bacteria like *Legionella pneumophila* manipulate host ubiquitin signaling through DUBs such as LotA, which specifically removes K6-linked ubiquitin chains. This activity protects the Legionella-containing vacuole from ubiquitin-dependent degradation by preventing the accumulation of K6-linked ubiquitin chains and VCP recruitment for its clearance [56,57]. In contrast to this, upon viral infection, IFN regulatory factor 3 (IRF3) is modified with K6-linked ubiquitin chains that promote its binding to type I IFN gene promoters and boost antiviral immunity. OTUD1 removes these chains, causing IRF3 to detach from DNA and suppressing IFN transcription without affecting IRF3’s stability or localization [58]. . Although K6-linked ubiquitin chains can mediate proteasomal degradation in some contexts, they are not exclusively degradative. Emerging evidence indicates that they also perform broader regulatory functions, in particular, K6 linkages have been shown to stabilize proteins [46,59,60] and participate in multiple non-degradative cellular functions [61,62], underscoring their expanding functional repertoire.

### K11-linked chains

The K11-linked ubiquitin chains are generally associated with maintenance of cell cycle and protein degradation. APC/C (Anaphase promoting complex/cyclosome) is a multi-subunit E3 RING ligase that controls cell-cycle progression [63] and preferentially tags its substrates with K11-linked ubiquitin chains [64,65]. In fact, inhibiting APC/C activity significantly reduces the formation of K11-linked ubiquitin chains [66], while inhibiting the proteasome activity accumulates K11-linked ubiquitin chains [67], indicating that APC is the primary source of K11 linkages in cells. Different E2s play in concert to form long ubiquitin conjugates on APC substrates. For example, UbE2S extends the chains by adding K11 linkages to substrates already ubiquitinated by UbcH10 or UbcH5 [64,68], facilitating mitotic exit after prolonged spindle assembly checkpoint activation [68,69]. RBX2, a RING E3 ligase known to associate with CUL5, also utilizes UbcH10 and UbE2S to mediate the K11-linked ubiquitination of β-TrCP1 and makes it amenable for degradation [70]. Interestingly, RBX2 and APC compete with each other for UbE2S binding, to modulate the G2-to-M transition. This antagonistic interaction likely serves as a regulatory mechanism to maintain cell cycle fidelity [71]. UbE2S also promotes the addition of K11-linked ubiquitin chains on a RING E3 ligase, VHL, leading to its proteasomal degradation and promotion of glycolysis in hepatocellular carcinoma [72].

Cellular inhibitor of apoptosis 1 (c-IAP1), a RING-type E3 ligase, along with UbcH5, facilitates the addition of K11-linked ubiquitin chains on receptor-interacting protein 1 (RIP1), which is crucial for TNFα-induced NF-κB activation. As NF-κB essential modifier (NEMO) effectively binds K11-linked ubiquitin chains, this linkage also plays a signaling role in promoting proliferative cellular responses [73]. In addition, K11 linkage was demonstrated to have roles in mitophagy and mitochondria quality control. A cytosolic RING E3 ligase MGRN1 trans-ubiquitinates the endoplasmic reticulum RING E3 ligase GP78 via K11 linkages to down-regulate mitophagy and regulate mitochondrial homeostasis [74]. On the other hand, K11 linkage has also been implicated in Hedgehog signaling. Ter94 ATPase facilitates the partial proteasomal degradation of Ci ubiquitinated by the Cul1-Slimb E3 ligase complex. This E3 ligase primarily attaches K11-linked ubiquitin chains to Ci, indicating that both Ter94 and K11-linked ubiquitination play key roles during Hedgehog signaling [75].

While K11 linkage largely targets protein towards degradation, there is no change in the stability of a F-box protein AnkB from *Legionella pneumophila* upon K11-linked ubiquitination by the host RING E3 ligase Trim21 [76]. In another study, a RING E3 ligase, RNF26, catalyzes K11-linked ubiquitination of STING, which prevents its degradation and consequently amplifies the production of type I interferons and proinflammatory cytokines [77]. Similarly, K11-linked ubiquitination of β-catenin mediated by the HECT E3 ligase, EDD and UbE2S shown by independent groups also stabilize the protein and promote Wnt signaling and cancer progression [78,79]. This suggests that there can be additional roles for K11-linked ubiquitination beyond degradation. E3 ubiquitin ligases often operate in conjunction with de-ubiquitinases, and their antagonistic activities are tightly co-ordinated to preserve cellular homeostasis. Removal of K11-linked ubiquitin chains on Beclin-1 by USP19 promotes autophagy [80]. Similarly, USP8 removes K11-linked chains from SQSTM1/p62 (sequestosome 1) to suppress autophagy [81]. The OTU family de-ubiquitinase Cezanne exhibits high specificity for K11-linked ubiquitin chains [82], and de-ubiquitinates APC/C substrates during mitosis to maintain chromosome stability [83]. Additionally, it stabilizes HIF-1α by removing K11-linked chains, thereby protecting it from degradation via chaperone-mediated autophagy [84].

### K27-linked chains

The K27-linked ubiquitin chains hold a variety of functions which determine the fate of the protein. While some evidence suggests K27 linkage leads to protein degradation, it is also involved in other functions like DNA damage, protein secretion, cell adhesion, etc. For example, a K27-linked autoubiquitination of Arkadia RING E3 ligase hampers its protein stability and leads to proteolysis [85]. Besides proteasome degradation, K27 linkages are also involved in autophagy and lysosome-mediated degradation [86–90]. On one hand, LATS1-induced K27 ubiquitination on Beclin1 stabilizes the protein and inhibits autophagy [86], whereas several examples involving RING E3 ligases like RNF185-mediated K27 linkage of Ebolavirus (EBOV) glycoprotein GP_1,2_ [87], MARCH8-mediated ubiquitination of MAVS [88], and autoubiquitination of TRIM23 [89] promote autophagy. In addition, RING E3 ligase TRIM21 catalyzes K27-linked ubiquitination of UFD1 and prevents its assembly into the VCP/p97 complex. This impairs the ERAD substrate degradation and triggers a pro-apoptotic unfolded protein response [91]. VDAC1 (voltage-dependent anion channel 1) is a mitochondrial substrate of a RBR E3 ligase, Parkin, which mediates its K27-linked polyubiquitylation to promote mitophagy [92]. Moreover, K27-linked ubiquitin chain conjugates on Jun mediate lysosomal localization via ubiquitin readers like HRS and TSG101 and lead to the lysosomal degradation of ubiquitinated Jun [90]. Therefore, K27-linked ubiquitination participates in different modes of degradative pathways.

Apart from degradative fate, K27 linkages are involved in other cellular processes. A RING E3 ligase, RNF168, mediates K27-linked ubiquitination of H2A family histones, which is essential for the activation of DNA damage response [93]. Also, HECT E3 ligase, HACE1-mediated K27-linked ubiquitination was demonstrated to promote the protein secretion of a transcription factor YB1 [94], and fibronectin which influences cell adhesion and migration [95]. During viral infection, K27-linked ubiquitination is also involved in the localization of host STING-TBK1 ubiquitinated complex from ER to perinuclear microsomes for TBK1 activation [96], as well as ubiquitination of viral proteins NS2B3 protein complex [97], suggesting the role of K27-linked ubiquitin chains in antiviral response and innate immune signaling. Additionally, K27-linked ubiquitination was shown to promote cell proliferation as HECT E3 Itch mediated K27-linked ubiquitin chains on BRAF [98] and TIEG1 [99], activates MAPK pathway and tumor progression.

### K29-linked chains

An affimer-based chemical screen for recognizing K29-linked ubiquitin chains revealed that this linkage plays a role in cellular responses to proteotoxic stress and in regulating the cell cycle [100]. The colocalization of VCP—but not the proteasome—with K29-linked ubiquitin chains suggests a non-degradative role for this modification [100]. Cullin 4-based E3 ligases mediate nonproteolytic K29-linked ubiquitination of the influenza A virus PB2 replication protein, supporting viral replication [101]. During viral infection, the F-box protein FBXO21, part of the SCF E3 ligase complex, mediates K29-linked ubiquitination of ASK1 (Apoptosis Signal-regulating Kinase 1), promoting its activation and subsequent induction of type I interferons in antiviral response [102]. In addition, K29-linked ubiquitination of Hepatitis B core (HBc) protein also plays a role in HBV infection cycle [103].

Further, the role of K29 linkage in Wnt/β-catenin signaling is demonstrated, although in a context-dependent manner. On one hand, HECT E3 ligase EDD promotes nuclear accumulation and up-regulation of both GSK-3β and β-catenin by stabilizing β-catenin through K29-linked ubiquitin chains and activates Wnt signaling [78]. On the other hand, Smurf1, also a HECT E3, catalyzes non-proteolytic K29-linked ubiquitin chains on Axin which leads to suppression of Wnt signaling [104]. Moreover, K29 linkage has also been implicated in Notch signaling, where in contrast to their stabilizing role, addition of K29-linked ubiquitin chains by Itch, a HECT E3 ligase on Deltex (DTX), a RING E3 ligase and Notch receptor leads to lysosomal degradation [105,106]. Additionally, K29-linked ubiquitin fusions on β-galactosidase and dihydrofolate reductase mark them for degradation in yeast [107]. Identification and characterization of a K29 selective de-ubiquitinase, TRABID [108,109] revealed that K29-linked ubiquitin chains often occur as part of mixed or branched polyubiquitin structures. Notably, short K29 linked chains (diUb to tetraUb) are frequently embedded within complex polyubiquitin assemblies [110]. Though K29-linked ubiquitination is a versatile modification involved in diverse cellular functions, its mechanisms and biological roles—particularly in protein degradation and beyond—remain incompletely understood and require further investigation.

### K33-linked chains

K33-linked ubiquitination is among the least understood ubiquitin linkage types, with limited studies exploring its physiological roles. It is generally considered non-degradative, as it does not accumulate with proteasomal inhibition [61]. Co-operation between a HECT E3, Itch, and a RING E3, Cbl-b, together conjugate a K33-linked ubiquitin chain on TCR-ζ to regulate the cell surface receptor-mediated signal transduction [111]. In another example, Nrdp1, a RING E3 ligase, catalyzes K33-linked ubiquitin chain on Zap70 to attenuate early TCR signaling [112]. Cul3-KLHL20 mediates a nondegradable K33-linked ubiquitination of coronin 7 (Crn7), promoting its recruitment to the trans-Golgi network (TGN) via Eps15 interaction. This modification is essential for TGN-associated F-actin assembly and post-Golgi transport carrier formation [113]. Further, HECT E3 ligase, HECTD3, mediates K33-linked ubiquitination of PKR to enhance RNA virus replication and simultaneously trigger a delayed inflammatory response [114]. During infection with DNA and RNA viruses, TBK1 undergoes K33-linked ubiquitination, which promotes IRF3 activation and antiviral signaling. USP38 reverses this modification, leading to TBK1 degradation via the proteasome and attenuation of the antiviral response [115].

### K48-linked chains

K48-linked ubiquitin chains, the most studied linkage, serve as the primary proteolytic signal, which marks the substrates for recognition and degradation by the 26S proteasome [116]. The foundational discovery linking ubiquitin to protein degradation emerged from studies on ATP-dependent proteolysis in rabbit reticulocytes [117,118]. The identification of ATP-dependent proteolytic factor 1 (APF-1), which was later recognized as ubiquitin, a highly conserved, heat-stable polypeptide that tags proteins for degradation [119]. Soon after, pivotal work revealed that K48-linked ubiquitin chains serve as the primary signal directing substrates to the proteasome [120], offering the first glimpse into the now intricate *ubiquitin script*. One of the earliest insights into K48-linked ubiquitin chains as signals for proteasomal degradation came from studies on the cyclin-dependent kinase inhibitor Sic1, which is ubiquitinated by the SCF^Cdc4^ E3 ligase complex and the E2 enzyme Cdc34 [121]. Ubiquitin readers such as the Cdc48 cofactor Npl4 exhibit selectivity for K48-linked ubiquitin chains, thereby facilitating the recruitment of ubiquitinated substrates to the proteasome for degradation [122]. K48-linked ubiquitin chains accumulate at DNA damage sites, where they promote the proteasomal degradation of JMJD2A, JMJD2B, and L3MBTL1 via RING ligases, RNF8/RNF168, facilitating 53BP1 recruitment on DNA lesions [123]. Structurally, K48-linked ubiquitin chains adopt a compact, ‘closed’ conformation in which adjacent ubiquitin molecules interact with each other (Figure 3F) [124]. This structural arrangement allows specific recognition by proteasomal receptors, ensuring efficient targeting and degradation of ubiquitinated substrates [125,126].

While K48 linkages largely target the substrates for proteasomal degradation, however, in combination with K63 linkage, they target low-density lipoprotein receptor (LDLR) for lysosomal degradation [127]. Interestingly, HECT E3 HACE1 mediated ubiquitination of OPTN with K48-linked ubiquitin chains, enhances its interaction with p62/SQSTM1 to form the autophagy receptor complex and promotes autophagy-dependent degradation [128]. An exception to its canonical proteolytic role, K48-linked autoubiquitination of the plant E3 ligase DA2 does not affect its stability but instead modulates its E3 ligase activity, highlighting a regulatory function of this linkage beyond degradation [129]. MINDY1 is a highly selective de-ubiquitinase which prefers binding with K48-linked polyubiquitin chains [130]. Otubain 1 (OTUB1), a DUB belonging to OTU family, has specificity towards cleavage of K48-linked ubiquitin chains [131], as its de-ubiquitination on FOXM1 facilitates tumor progression and predicts a poor prognosis in ovarian cancer [132].

### K63-linked chains

K63 linkage levels remain largely unchanged upon proteasome inhibition, supporting their established role in nonproteolytic functions such as stress responses, signaling, trafficking, cell adhesion and migration, and autophagy [133–135]. K63-linked ubiquitin chains play a key role in regulating endocytosis and membrane protein trafficking. They act as molecular tags that direct transmembrane proteins, such as the epidermal growth factor receptor (EGFR), to be internalized from the plasma membrane and sorted into the intraluminal vesicles of multivesicular bodies (MVBs) [136–138]. These MVBs mature from early endosomes and eventually fuse with lysosomes, where the cargo is degraded [139]. This sorting is facilitated by the ESCRT complexes, which contain ubiquitin-binding domains that specifically recognize ubiquitinated cargo [140,141]. Beyond endocytosis, K63-linked ubiquitin chains are also implicated in autophagy by engaging receptors like p62 and NBR1, which have UBDs with K63 specificity [142–145]. E3 ligases such as RBR E3, Parkin, and RING E3, TRAF6 generate these chains, and they are found on selective autophagy cargoes [146,147]. K63-linked ubiquitin chains on core autophagy regulators, ULK1 [148] and Beclin1 [149], further promote autophagosome formation and autophagy initiation. Thus, K63-linked ubiquitin chains serve as versatile signals for both endolysosomal degradation and selective autophagy [150]. With regard to cellular localization, K63-linked ubiquitination of RagA GTPase is mediated by lysosome-anchored RING E3 ubiquitin ligase RNF152 to negatively regulate mTORC1 signaling [151]. Additionally, to mediate the translocation of BNIP1 from ER to mitochondria, RING E3 ligase RNF186 catalyzes K63-linked ubiquitination of BNIP1 [152]. Subsequently, RING E3 RNF185 further conjugates K63 linkages to BNIP1 and enables the recruitment of the autophagy receptor p62 to facilitate autophagosome formation [153]. K63-linked ubiquitination plays a critical role in DNA repair, as the SCF^FBXW7^ E3 ligase is recruited to DNA double-strand breaks to catalyze K63-linked ubiquitination of XRCC4, thereby facilitating efficient non-homologous end joining (NHEJ) [154]. In the context of viral infection, on one hand, K63-linked ubiquitination of Hepatitis C Virus NS 2 protein enhances the envelopment of virus and promotes its infection [155]. On the other hand, K63-linked ubiquitination inhibits HIV-1 reverse transcription and disassembles the capsid formation [156]. K63-linked ubiquitination has been specifically observed in the inclusion biogenesis; for example, disease-associated Tau and SOD1 positive inclusions are enriched with K63-linked ubiquitin chains [157]. In another scenario, HECT E3, WWP2-mediated K63-linked ubiquitination of Dvl2 is essential for its phase separation into condensates, which activates Wnt signaling [158].

Interestingly, K63-linked ubiquitin chains have been selectively implicated in the formation of protein inclusions in Huntington’s, Alzheimer’s, and Parkinson’s diseases [159], suggesting that under pathological conditions, the proteasome may also contribute to the degradation of K63-linked substrates [160,161]. In fact, TGF-β induces K63-linked ubiquitination of EZH2 and promotes its proteasomal degradation during biliary fibrosis [162]. Also, the addition of K63-linked ubiquitin chains on TXNIP1 enables it for proteasomal degradation by extending K48 linkages and thus, acting as a mark for degradation in regulating cellular fate [163]. K63-linked ubiquitin chains exhibit unique structural properties (Figure 3G), where distinct conformational states mediate specific interactions [164,165]. Their extended, open conformation enables them to act as scaffolds that recruit signaling complexes via ubiquitin-binding domains [124], such as binding with tandem UIM of RAP80 during DNA damage repair; while the C2 closed state interacts with the NZF domain of TAB proteins to activate NF-κB signaling; and the C1 closed state engages the ZnF4 domain of A20 to terminate NF-κB signaling [166].

DUBs which specifically cleave K63-linked ubiquitin chains are AMSH which rescues cargo from lysosomal degradation [138], USP4 which regulates the activation of splicing [167], and CYLD which negatively regulates the key signaling pathways involved in inflammation, immune responses, cell survival, and tumorigenesis [168]. Together, K63-linked ubiquitination functions as a scaffold for protein–protein interactions, regulates signaling pathways and modulates protein localization and activity.

### M1-linked chains

Till date, the only M1 linkage‐specific E3 ligase system exists which is called the linear ubiquitin chain assembly complex (LUBAC) [169]. LUBAC comprises RBR E3 ligases HOIL1‐interacting protein (HOIP, also known as RNF31), hem-oxidized IRP2 ubiquitin ligase 1L (HOIL‐1L), and Shank associated RH‐domain interacting protein (SHARPIN) [170]. The ligase complex generates M1-linked ubiquitin chains via head-to-tail linkages between the C- and N-termini of ubiquitin, rather than conventional lysine residues, highlighting a unique capacity to assemble linear ubiquitin chains. LUBAC activates the canonical NF-κB pathway by binding NEMO and conjugating M1-linked ubiquitin chains onto specific lysine residues of NEMO, facilitating downstream signaling [171]. Moreover, XIAP and LUBAC constitute essential ubiquitin ligases in NOD2 signaling in inflammation and innate immunity [172]. Linear ubiquitination also contributes to the pathogenesis of optineurin (OPTN)-associated amyotrophic lateral sclerosis (ALS), as OPTN relies on binding to linear ubiquitin chains to regulate NF-κB signaling and apoptosis [173]. While OTULIN is a DUB which specifically hydrolyzes M1 linkages [174], CYLD has similar affinities for both K63 and M1 linkages in cells [175]. *Legionella pneumophila* also expresses the effector protein RavD, a highly specific de-ubiquitinase for M1-linked ubiquitin chains. RavD removes M1-linked ubiquitin chains from Legionella-containing vacuoles, thereby suppressing NF-κB signaling during infection [176]. It remains an open question whether M1-linked ubiquitination has broader functional roles beyond those currently known, and whether E3 ligases other than LUBAC can mediate this modification.

### Mixed-linkage chains

Mixed-linkage, or heterotypic, ubiquitin chains consist of the conjugation of multiple ubiquitin molecules linked through different lysine residues or methionine, but with each ubiquitin modified at only one site. The specificity of ubiquitin chain formation is encoded in the E2:E3 pairing. A single E3 can recruit multiple E2 enzymes with distinct linkage preferences to generate heterotypic chains, while some E3s can also assemble heterotypic chains using a single E2 [177]. Though individual homotypic linkages have a dedicated functional outcome of their own, they can either compete or facilitate other linkages to segregate different fates of the protein. For example, HECT E3 ligase NEDD4 enhances Beclin1 stability and autophagy via K6/K27-linked ubiquitination while preventing K48-linked degradation [178]. TRAF6-mediated mixed-linkage of K6, K27, and K29-linked ubiquitin chains on DJ-1 and α-synuclein (aSYN) promotes their aggregation into Lewy bodies in Parkinson’s Disease [179]. The atypical ubiquitination chains involving K6/K27/K29 linkages mediated by MID1 are also involved in the proteasome-mediated degradation of BRAF35 [180]. In Plasmodium, food vacuole-associated enolase undergoes atypical ubiquitination involving M1-linked and K6-linked chains [181]. The viral RING E3 ligase MIR2 from Kaposi sarcoma-associated herpesvirus forms mixed K11/K63-linked ubiquitin chains, which serve as internalization signals for MHC I by recruiting Epsin1, a ubiquitin-binding adaptor protein [182].

Using linkage-specific affimers, three human E3 ligases—RING E3 RNF144A, RBR E3 RNF144B, and HECT E3 HUWE1—were demonstrated to be capable of assembling mixed ubiquitin-linked chains composed of K6, K11, K48, and K63 linkages [51]. During PINK1/Parkin-mediated mitophagy, Parkin also builds K6-K11-K48-K63-linked ubiquitin chains on outer mitochondrial membrane (OMM) proteins to signal their clearance [47,183]. Such combinations of linkages can act as powerful degradation tags. Just as E2s exhibit specificity for forming certain ubiquitin linkages, DUBs also show linkage specificity. The NZF1 domain of the de-ubiquitinase TRABID specifically recognizes and binds K29- and K33-linked diUb, enabling selective cleavage of these linkages [184]. USP21 acts as a key de-ubiquitinase for STING, suppressing DNA virus-induced type I interferon production by removing K27- and K63-linked ubiquitin chains from STING during HSV-1 infection [185]. Similarly, USP19 negatively regulates TNF-α and IL-1β-induced NF-κB signaling by specifically removing K63- and K27-linked ubiquitin chains from TAK1, thereby impairing TAK1 activity, disrupting its interaction with TAB2/3, and dampening the inflammatory response [186]. These distinct linkages confer specific ubiquitin conformations suggesting recruitment of different molecular machineries for diverse cellular functions, which can significantly enhance the specificity, precision, and functional diversity of ubiquitin-mediated signaling pathways.

### Branched-linkage chains

Branched-linkage ubiquitin chains are characterized by a single ubiquitin molecule modified at multiple lysine residues or methionine, resulting in a bifurcated structure. Their synthesis requires either enzymes with relaxed linkage specificity or the co-ordinated action of enzymes with distinct acceptor site preferences [187]. Branched-linkage ubiquitin chains can either protect substrates from degradation or, conversely, prevent de-ubiquitination and promote their degradation in a context-dependent manner. For example, in Alzheimer’s, PHF-Tau is mainly K48-ubiquitinated for degradation, but co-modification with K6/K11 impairs the proteasomal degradation and contributes to the accumulation of degradation-resistant Tau aggregates [188]. In another example, the E4 chain elongation factor Ufd2p catalyzes K48-linkages on pre-existing K29-linked chains built by HECT E3 ligase Ufd4p, generating branched-linkage chains. This ‘linkage switching’ is crucial for directing substrates, originally marked with non-degradative K29-linked chains, to the proteasome and is essential for ERAD and stress responses in yeast [189]. This K29/K48-linked ubiquitination duo is also catalyzed co-operatively by the HECT E3 Ufd4 and RING E3 Ubr1 and serves as an enhanced degradation signal. Ufd4 extends K48-linked ubiquitin chains with K29 linkages, thereby enhancing polyubiquitination and promoting efficient proteasomal targeting [190,191]. Similarly, K11/K48-branched-linkage ubiquitin chains act as enhanced and efficient signals for proteasomal degradation [192]. Mechanistically, recognition of specific ubiquitin chains typically requires simultaneous engagement of at least two ubiquitin units by binding motifs positioned to favor a particular chain topology; for branched-linkage chains, this often involves recognition of three ubiquitin moieties at once [193]. This occurs because RPN1, a proteasomal lid subunit and ubiquitin receptor with multiple binding surfaces, recognizes both the hydrophobic patch and an α-helical segment of ubiquitin, resulting in a ~ 15 fold higher affinity for K11/K48-branched trimers compared with K48-linked dimers, and a ~ 4 fold higher affinity relative to K11-linked dimers [194]. This enhanced affinity is strictly dependent on branching, as linear trimers containing sequential K11 and K48 linkages are bound far less efficiently than the branched species. This mode of recognition provides a mechanistic basis for the enhanced proteolytic potency of branched ubiquitin linkages.

In contrast, while K11 linkage alone typically signals for degradation, its combination with K29 and K63 linkages on p53 instead stabilizes and activates p53 in response to DNA damage [195]. Even branched ubiquitin chains composed of identical linkage types can vary in their architecture, due to differences in the branch number, position, or sequence which can influence the functional outcome of the modification [15]. While K48-linked chains of length ≥ 3 are targeted by proteasome for degradation, K63-linked chains, regardless of length, are rapidly cleaved and do not trigger degradation. Interestingly, the position of linkage in branched K48/K63 chains determines the fate: if K48 is substrate-proximal, degradation occurs; if K63 is proximal, the chain is quickly disassembled, neutralizing any degradative signal from a distal K48 branch [196,197]. Although the mechanism of ubiquitin chain branching remains unclear, it is likely that an intrinsic noncovalent ubiquitin-binding site within the E3 ligase helps position the internal acceptor ubiquitin to enable branching [198]. However, how this site is regulated to ensure timely and spatially appropriate branching is unknown. Moreover, in most cases, it is still unclear how branch point ubiquitins are selected or how many branch points exist within a chain. While proteomic approaches for identifying ubiquitin chains have advanced substantially, the evidence supporting certain branched chain types remains comparatively weak. This is largely due to limitations in analytical resolution, where multiple mixed-linkage chain conformations can masquerade as branched species, creating an apparent but not definitive signal for true branching.

### Unanchored polyubiquitin chains

Ubiquitin can form chains not linked to any substrate, known as free or unanchored chains. These chains are recognized as active players in immune signaling and stress responses, with distinct physiological roles in ubiquitin-mediated processes [199]. Unanchored K63-linked ubiquitin chains critically regulate the canonical NF-κB pathway. By binding to the NZF domain of TAB2, these chains promote clustering of TAK1 complexes and enable their mutual phosphorylation and subsequent NF-κB activation [200]. Conversely, the same chains can dampen this pathway through interaction with A20, a dual-function enzyme with DUB and E3 ligase activities, which competes with TAB2 for ubiquitin binding. A20 forms a complex with NEMO on the polyubiquitin scaffold, prevents TAK1-mediated phosphorylation of IKK and subsequent inhibition of NF-κB signaling [201]. Unanchored polyubiquitin chains also play a role in aggresome clearance. The proteasomal DUB POH1 releases free ubiquitin chains by cleaving ubiquitinated proteins. The K63-linked unanchored chains bind and activate HDAC6, which facilitates autophagy-mediated processing of aggresomes [202]. Unanchored ubiquitin chains can be regulated through multiple mechanisms. A conventional route could be their disassembly into mono-ubiquitin for reuse. Alternatively, they may be degraded directly, or conjugated *en bloc* onto substrates, or sequestered by ubiquitin-binding proteins as a reserve pool for ubiquitination during energy-demanding conditions and may help conserve cellular ATP [199].

### Signal decoding of ubiquitin chains

#### Readers of ubiquitin chains

Different ubiquitin chain linkages display characteristic conformational preferences (shown in Figure 3) with some adopting compact structures stabilized by inter-ubiquitin contacts (such as K48-linked chains), whereas others sample more extended and flexible conformations (such as K63-linked chains) (Figure 3F and G) [124]. These structural features influence their recognition and processing by ubiquitin-binding domain (UBD) proteins [203]. These ubiquitin ‘readers’ or ‘decoders’ interpret the chain’s topology and length to direct specific cellular outcomes [204]. UBDs are categorized into nearly 25 families based on their structural folds, broadly classified into helical (e.g., Ub associated; UBA, Ub interacting motif; UIM), zinc finger (ZnF), ubiquitin-conjugating-like, pleckstrin homology (PH), among others) [204]. While most UBDs recognize the canonical hydrophobic patch on ubiquitin’s β-sheet surface centered around Ile44 and Val70, others engage non-canonical regions such as the C-terminal di-Gly, the Asp58-centered polar surface, or alternative hydrophobic patches near Leu8 or Ile36 [205]. Specific ubiquitin–UBD interactions are key regulators of diverse cellular processes, including protein stability, receptor trafficking, DNA damage response, and inflammation [206]. For example, UBDs in Y-family polymerases are essential for DNA translesion synthesis via binding to monoubiquitinated PCNA [207]. Additionally, UBD proteins such as Epsins, Hrs, and Vps9 act as adaptors that connect ubiquitinated cargo to the clathrin-mediated sorting machinery at the plasma membrane and endosomes [208].

Ubiquitin chain linkages are not static structures but are flexible ensembles of multiple conformations that facilitate recognition by specific UBDs leading to distinct functional outcomes of ubiquitination [165,206,209,210]. This structural flexibility can enable different linkage types to be recognized by the same UBD, or conversely, allow a single chain type to interact with multiple UBDs [124,211]. Such conformational plasticity represents an important mechanistic feature of the *ubiquitin script* and contributes to the functional diversity of ubiquitin signaling within the cell. For example, RAD23A, a UBD protein involved in targeting substrates to the 26S proteasome, prefers binding with K48-linked chains with 3.6-fold higher affinity than K63-linked chains, and 70-fold higher affinity than monoubiquitin [212,213]. Contrastingly, structural comparisons of TAB2-NZF bound to K6- and K63-linked diUb reveal a shared binding mechanism, where flexibility in the C-terminal region of the distal ubiquitin allows TAB2-NZF to accommodate both linkages, thereby underpinning its dual specificity to both K6 and K63-linked ubiquitin [214–216]. In another scenario, despite their structural similarity, chain types such as K63- and M1-linked ubiquitin (Figure 3G and H) adopt conformations that can be selectively recognized by specific UBDs [217]. For instance, the UBAN domain of NEMO binds M1-linked diUb with much higher affinity than K63-linked chains because it can simultaneously engage both ubiquitin moieties and the linear linker in M1 linkages, whereas it interacts with only one ubiquitin in K63 chains [218–220]. Conversely, the NZF domain of TAB2 preferentially binds K63-linked diUb through tandem interactions with adjacent ubiquitins, a configuration that is not supported by M1 linkages, illustrating how subtle structural differences can dictate linkage-specific recognition and downstream signaling outcomes [221].

#### Ubiquitin chain editing

Ubiquitin chain editing represents one of the most intricate ways in which the ubiquitin script is employed. In this process, an existing ubiquitin chain on a substrate is remodeled or replaced with a different chain type, thereby altering the substrate’s downstream fate [14]. This editing is often facilitated by the co-ordinated action of DUBs and E3 ligases. Ubp2 and Ubp3 DUBs are essential for clearing cytosolic misfolded proteins following heat shock by facilitating their proteasomal degradation. They achieve this by removing non-degradative K63-linked ubiquitin chains and enabling HECT E3 Rsp5 to assemble K48-linked chains that signal for proteasomal targeting [222]. This editing of ubiquitin chain topology is crucial for maintaining cytosolic protein quality under stress. In another scenario, A20, a unique enzyme possessing both DUB and E3 ligase functions, acts as a negative regulator of NF-κB signaling. A20 first removes K63-linked ubiquitin chains from RIP and attaches K48-linked chains to it, marking it for proteasomal degradation [223]. This dual activity allows A20 not only to terminate K63-dependent signaling but also to clear signal transducers from the pathway, preventing immediate reactivation and ensuring tight control over NF-κB signaling. Several additional cases have shown co-ordinated activity between E3 ligases and DUBs, where their combined action edits ubiquitin chain types on substrates to fine-tune the functional outcome of ubiquitination [184,224,225].

#### How long do ubiquitin chains need to be?

Another important factor influencing selective recognition by ubiquitin-binding proteins is the length of the ubiquitin chain. Longer K29- and K33-linked chains (e.g., hexamers and above) tend to associate more with metabolite interconversion enzymes such as hydrolases, transferases, and oxidoreductases [226]. In contrast, shorter chains (e.g., di- and tetraubiquitin) preferentially bind protein-modifying enzymes like kinases, proteases, and ubiquitin ligases [226]. Thus, chain length plays a critical role in determining the specificity of ubiquitin–UBD interactions. Together, UBDs decode the diversity of ubiquitin chains through multivalent interactions, sequence context, and conformational adaptability, enabling linkage-specific recognition. The functional outcome of substrate ubiquitination is shaped by how ubiquitin receptors, containing both ubiquitin-binding and localization domains, engage with their targets. These co-ordinated interactions not only interpret the ubiquitin signal but also direct substrates toward specific cellular fates such as degradation, localization, or integration into signaling complexes [14].

#### Analytical methods to identify ubiquitin chain types

The rapid expansion of the ubiquitin-chain field has been driven by new analytical tools that can precisely detect and discriminate between linkage types. Advances in mass spectrometry, chain-specific antibodies, and engineered ubiquitin probes have enabled high-resolution mapping of ubiquitin architectures that were previously inaccessible. Here we are discussing a few methods briefly.

#### Affinity-tagged ubiquitin

Affinity-tagged ubiquitin approaches rely on expressing tagged ubiquitin in cells and enriching ubiquitin–substrate conjugates by affinity chromatography. Tags such as His, Strep, or AviTAG on the N-terminus of ubiquitin allow identification of homotypic [20,227–229] and heterotypic chains (e.g., K11/K27, K27/K29, K29/K33) [230]. Utilizing a lysine-less ubiquitin (Ub K0) can also help in detecting mono-ubiquitinated substrates [231]. Although these methods mainly enrich lysine-linked chains, a recent strategy specifically isolates M1 chains. By introducing a Strep-II tag at Ub R54 and mutating all lysines to arginine, the M1 linkage can be studied [232]. Together, these tools enable stringent enrichment of covalently attached ubiquitin chain types. However, overexpression of tagged ubiquitin can cause artificial ubiquitination and variable incorporation of the tagged ubiquitin into substrates or chain types and may introduce substantial analytical bias.

#### Proteomics

Trypsin digestion of ubiquitin-conjugated proteins generates a signature peptide in which a di-glycine (Gly–Gly) remnant from ubiquitin remains attached to the modified lysine, producing a characteristic 114.1 Da mass shift and a missed cleavage at that site [20]. Each ubiquitin-linked chain generates unique diGly signatures, which makes it easier to identify chain types using proteomics [20]. Antibodies against the diGly remnant are utilized to enrich the diGly peptides, and when combined with tandem mass tag (TMT) labeling, enable quantitative estimation of ubiquitination. Although widely used and unbiased for lysine ubiquitination, this method does not capture non-lysine ubiquitination events such as serine-, threonine-, or cysteine-linked modifications [233].

The development of Ubiquitin Absolute Quantification (Ub-AQUA) heavy reference peptides for all seven isopeptide linkages, as well as the M1 linkage, allowed the precise quantification of ubiquitin linkage stoichiometry on substrates [234]. These standards were either generated *in vitro* by specific E3 ligases or isolated from cells or tissues using high-resolution mass spectrometry [235]. Despite its broad utility in dissecting ubiquitin signaling, its conventional format cannot quantify most branched chains, with the exception of neighboring branches such as K6/K11, K11/K27, and K27/K29 [236].

While Ub-AQUA can quantify K48/K63-branched chains, it cannot resolve higher-order ubiquitin assemblies or determine the length of individual chain types [236]; however, incorporating middle-down proteomics can help infer branched ubiquitin linkages as well as the chain lengths on distinct proteins. In this strategy, ubiquitin chains are treated either by Ub-clipping or trypsin to preferentially cleave the R74–G75 bond, and the branch points as well as the chain length can be inferred by comparing the ratio of Ub R74 with Ub R74-GG species [237,238]. Although fragmentation patterns of Ub R74 can identify certain linkages (such as K48 and K63), determining linkage composition within complex substrates that contain multiple chain types remains challenging, especially since many targets are modified by several E3 ligases with distinct chain architectures.

#### Antibodies

Linkage-specific antibodies that recognize K11, K48, K63, and M1 chains have become powerful tools for probing the roles of specific ubiquitin linkages in cellular pathways, and antibody-based enrichment remains widely used [66,239,240]. These antibodies have been developed for standard immunoprecipitation workflows, and more recently, bispecific antibodies have been engineered to selectively enrich K11/K48 [241], K63/M1 [242] ubiquitinated linkages. Complementing these tools, synthetic libraries of humanized nanobodies, including those specific for K48 and K63, provide small, easily expressed reagents with high purity and good linkage selectivity, which offers an alternative to conventional antibodies [243,244].

#### De-ubiquitinating (DUB) enzymes

The development of de-ubiquitinases (DUBs) with defined linkage specificity has enabled Ub Chain Restriction analysis (UbiCRest), in which ubiquitinated proteins are treated with selected DUBs to selectively remove one or more chain types while leaving others intact [245]. The resulting products can be analyzed using linkage-specific antibodies or Ub-AQUA–based proteomics. However, because DUB activities on mixed-linkage and branched chains are not yet fully characterized, the outcomes of their action on complex chain architectures can be difficult to predict [246]. Nevertheless, UbiCRest has successfully been used to identify homotypic K63-linked chains as well as M1/K63 mixed-linkage chains in independent studies [245,247].

#### Engineered Ub-binding probes

Affimers are engineered, antibody-like ubiquitin binders built on a 12 kDa cystatin-fold scaffold, from which linkage-specific binders can be selected [51,248]. These reagents show high linkage-specificity at the diUb level, but appropriate controls are essential in cellular applications to avoid off-target enrichment, as illustrated by the reported cross-reactivity of a K33 affimer with K11 diUb [51]. Despite such caveats, affimers faithfully detect longer ubiquitin chains and have been effectively used to delineate E3 ligase–specific linkage patterns [51].

Tandem ubiquitin‐binding entities (TUBEs), built from repeated UBA domains, bind polyubiquitin chains and enable efficient purification of polyubiquitinated proteins under native conditions [249]. By shielding ubiquitin chains from both proteasomal degradation and de-ubiquitination, TUBEs act as ‘molecular traps’ and are widely used to isolate endogenous ubiquitinated species [249]. Linkage‐specific variants, including K29-, K63-, K48-, and M1-selective TUBEs, further expand their utility [110,130,219,221,250,251]. Chain length can also be estimated using trypsin digestion in the presence of ubiquitin-chain protectors such as Ub-ProT or trypsin-resistant TUBEs (TR-TUBEs), which preserve substrate-attached chains from trypsin cleavage during mass spectrometry, allowing the remaining ubiquitin fragments to report on chain length [236].

Recently, ubiquitin-chain fluorescence sensors based on linkage-specific ubiquitin-binding domains (UBDs) have emerged as valuable tools for detecting distinct chain types. UBAN domains (Ubiquitin binding in ABIN and NEMO) enable selective detection of M1-linked chains, while UIM domains from RAP80 and NZF domains from TAB2 can distinguish K63-linked chains [252]. These UBD-based biosensors provide useful prototypes for tracking additional ubiquitin chain architectures and ubiquitin-like signals *in vivo*. Fluorescence-based probes have further expanded detection capabilities. The inducible PolyUb-FC system, which uses split Kusabira Green fluorescence complementation, allows direct visualization of chain-specific polyubiquitination in live cells, while remaining non-fluorescent for monoubiquitination [253]. Similarly, the fluorescein-labeled ThUBD (ThUBD-Flu) probe enables highly sensitive visualization of polyubiquitin signals and surpasses traditional ubiquitin antibody methods in accuracy and sensitivity [254].

#### Ubiquiton

Ubiquiton is an inducible, linkage-specific polyubiquitination platform that enables substrate-directed assembly of M1-, K48-, or K63-linked chains [255]. It integrates engineered linkage-specific E3 ligases with cognate ubiquitination sites on target proteins and is not restricted to degradative outcomes. The system exploits split-ubiquitin technology [256], in which the N-terminal (NUb) and C-terminal (CUb) halves of ubiquitin reassemble into a native-like fold only when brought together by substrate–E3 proximity, triggering DUB-mediated cleavage at Gly76 and enabling chain initiation and extension in a linkage-defined manner [255]. Depending on the linkage type, NUb and CUb are positioned on the E3 or substrate to ensure selective chain formation. Despite its versatility, Ubiquiton has its limitations, such as chain length can only be partially tuned (e.g., by modulating rapamycin levels), endogenous E3s may extend or branch chains with alternative linkages which may interfere with Ubiquiton’s recognition of the substrates, and nearby unintended proteins may also become modified [255]. Despite its limitations, Ubiquiton’s ability to direct linkage-specific polyubiquitination offers a powerful means to control protein–protein interactions and signaling, particularly in synthetic biology applications. In this way, its engineered ‘writers’ complement the expanding toolkit of ubiquitin readers, erasers, and other reagents designed to decode and manipulate the ubiquitin script.

#### Summary and future perspectives

**Figure 4:**
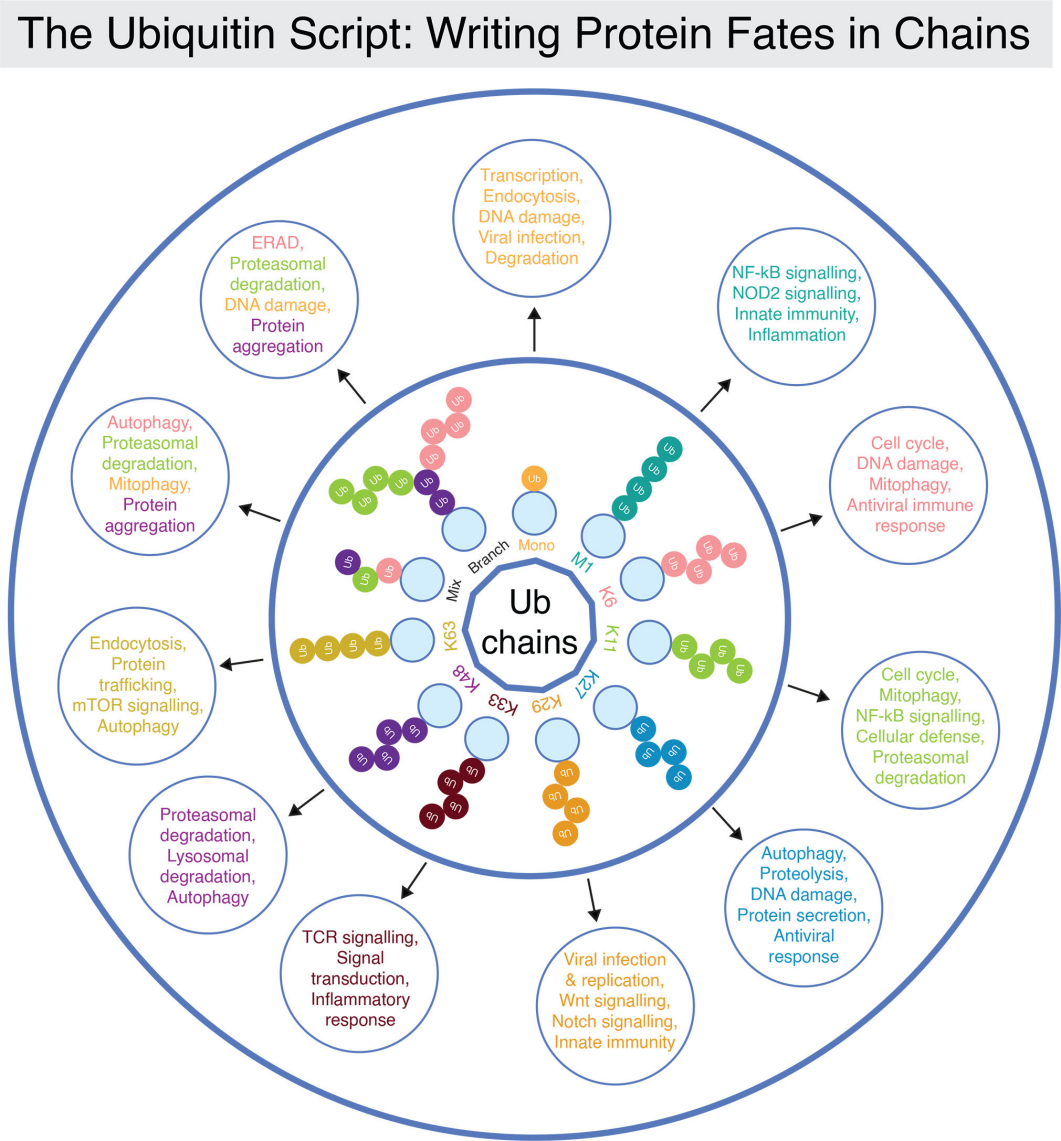
Topology matters.

In this review, we have attempted to distill the current understanding of ubiquitin linkage types and patterns, showcasing the remarkable complexity and versatility of this post-translational modification (shown in Figure 4 and Table 1). The diversity of ubiquitination arises from the variety of linkage types, branching architectures, and chain lengths, which together enable a vast array of signaling outcomes.

Ubiquitin chains with distinct linkages modulate substrate fate and influence diverse cellular pathways.

**Table 1:** Summarized information of ubiquitin linkages with examples.

Mono-ubiquitination			
Substrate	Modifier	Function	Mechanistic summary
Histone H2A, H2B	-	Transcription	Histone H2A, H2B monoubiquitination hinders the nucleosome folding and promotes multiple rounds of transcription [29,30]
GPCR, Maltose transporter, Galactose transporter Gal2p, and Pma1p	-	Endocytosis	Ligand-induced internalization from plasma membrane of a GPCR [31–34]
FANCD2	-	DNA damage	Monoubiquitination of FANCD2 promotes its localization at repair foci in Fanconi anemia [35]
β-galactosidase and α-globulin	-		Monoubiquitination of β-galactosidase and α-globulin leads to proteolytic degradation [36,37]
Orphan proteins	UBR4-KCMF1 ligase complex	Proteasome degradation	Monoubiquitination leads to degradation of orphan proteins [38]
**K6-linked chains**			
BRCA1/BARD1 Ub ligase	-	Essential for protein activity	K6-linked autoubiquitination of BRCA1/BARD1 is essential for its activity during S-phase and DNA damage [39,40]
Nucleophosmin, RPB8, CtIP	BRCA1/BARD1 Ub ligase	Cell survival, cell cycle, DNA damage	K6-linked ubiquitination of NPM, RPB8, CtIP is non-degradative [43–45]
α-tubulin	MGRN1	Mitosis	K6-linked ubiquitination of α-tubulin is essential for spindle orientation [46]
Mitochondria outer membrane proteins	E3: Parkin DUB: USP30, USP8	Mitophagy, Mitochondria quality control	K6-linked ubiquitination of MOM plays a role in mitophagy [47–50]
Mitofusin-2	E3: HUWE1	-	HUWE1 incorporates K6 linkages on Mitofusin-2 [51]
Ribosome-mRNA complex	E3: RNF14	Translation control	K6- and K48-linked ubiquitination on RNA-protein cross-links to regulate translation [53,54]
Viperin	E3: UBE4A	Antiviral response	K6 linkages on Viperin lead to its degradation and poor antiviral response [55]
IRF3	DUB: OTUD1	Antiviral immunity	K6 linkages of IRF3 boost antiviral immunity [58]
**K11-linked chains**			
-	APC/C E3 ligase complex, E2: UbE2S, UBCH10	Cell cycle progression	APC is the primary source of K11-linked degradative chains in cells [63–69]
β-TrCP1	E3: RBX2 E2: UBCH10, UbE2S	Cell cycle progression	K11-linked ubiquitination followed by proteolytic degradation of β-TrCP1 is crucial to regulate G2-M transition [70,71]
VHL	E2: UbE2S	Glycolysis	K11-linked ubiquitination of VHL leads to proteasomal degradation during HCC [72]
RIP1	E3: cIAP1, E2: UBCH5, UBD: NEMO	NF-κB signaling	K11-linked RIP1 ubiquitination is crucial for TNFα-induced NF-κB activation [73]
GP78	MGRN1	Mitophagy	GP78 ubiquitination via K11 linkage down-regulates mitophagy and regulates mitochondrial homeostasis [74]
Ci	Cul1-Slimb E3 ligase complex	Hedgehog signaling	K11-linked Ci ubiquitination plays key roles in Hedgehog signaling [75]
AnkB	E3: TRIM21	Cellular defense	K11-linked ubiquitination of AnkB is non-degradative [76]
STING	E3: RNF26	Inflammation	K11-linked ubiquitination of STING is non-degradative and promotes interferon signaling [77]
β-catenin	E3: EDD E2: UbE2S	Wnt signaling	β-catenin ubiquitination via K11 linkages stabilizes its levels and promotes Wnt signaling [78,79]
Beclin-1	DUB: USP19	Autophagy	Removal of K11-linked chains promotes autophagy [80]
SQSTM1	DUB: USP8	Autophagy	Removal of K11-linked chains suppresses autophagy [81]
HIF-1α	DUB: Cezanne	Autophagy	Removal of K11-linked chains prevents degradation [84]
**K27-linked chains**			
Arkadia	-	Proteolysis	K27-linked autoubiquitination of Arkadia E3 ligase leads to proteolysis [85]
Beclin1	-	Autophagy	LATS1 induced K11-linked ubiquitination stabilizes Beclin1 and inhibits autophagy [86]
EBOV glycoprotein GP1,2	E3: RNF185	Autophagy	Promotes autophagy [87]
MAVS	E3: MARCH8	Autophagy	Promotes autophagy [88]
TRIM23	-	Autophagy	TRIM23 autoubiquitination promotes autophagy [89]
UFD1	E3: TRIM21	ERAD	K27-linked ubiquitination of UFD1 prevents its degradation [91]
VDAC1	E3: Parkin	Mitophagy	K27-linked ubiquitination of VDAC1 promotes mitophagy [92]
Jun	-	Proteolysis	Lysosomal degradation [90]
H2A	E3: RNF168	DNA damage response	Non-degradative [93]
YB1	E3: HACE1	Protein secretion	K27-linked ubiquitination on YB1 leads to protein secretion [94]
Fibronectin	E3: HACE1	Protein secretion	K27-linked ubiquitination on fibronectin influences cell adhesion and migration [95]
STING-TBK1 complex	E3: AMFR	Cellular localization	Ubiquitinated STING-TBK1 localizes it from ER to microsomes [96]
NS2B3	-	Antiviral response	Innate immunity [97]
Braf, TIEG1	E3: ITCH	MAPK pathway	K27-linked ubiquitination activates MAPK pathways and tumor progression [98,99]
**K29-linked chains**			
PB2	Cul4 E3 ligase complex	Viral replication	Non-proteolytic ubiquitination of influenza A virus PB2 replication protein during viral infection [101]
ASK1	FBXO21	Antiviral response	K29-linked ubiquitination of ASK1 promotes its activation and induction of Type I interferons [102]
HBc	-	HBV infection	Hepatitis B virus infection cycle [103]
β-catenin	E3: EDD	Wnt signaling	Stabilizes β-catenin and activates Wnt signaling [78]
Axin	Smurf1	Wnt signaling	Suppression of Wnt signaling [104]
Deltex, Notch	E3: Itch	Notch signaling	Lysosomal degradation [105,106]
β-galactosidase, dihydrofolate reductase	-	Degradation	K29-linked ubiquitination leading to degradation [107]
**K33-linked chains**			
TCR-ζ	E3: ITCH, Cbl-b	Signal transduction	K33-linked ubiquitination of TCR-ζ regulates cell surface receptor-mediated signal transduction [111]
Zap70	E3: NRDP1	TCR signaling	Attenuation of TCR signaling [112]
Crn7	E3: Cul3-KLHL20	Endocytic trafficking	K33-linked ubiquitination of Crn7 is essential for Golgi-mediated transport [113]
PKR	E3: HECTD3	Inflammatory response	PKR ubiquitination enhances RNA virus replication and delays inflammatory response [114]
TBK1	-	Antiviral response	IRF3 activation and antiviral response [115]
**K48-linked chains**			
Sic1	SCF^Cdc4^ E3 ligase complex, E2: Cdc34	Proteasomal degradation	Degradative [121]
JMJD2A, JMJD2B, L3MBTL1	E3: RNF8, RNF168	DNA damage	Proteasomal degradation [123]
LDLR	-	Lysosomal degradation	Degradative [127]
OPTN	E3: HACE1	Autophagy	Autophagic degradation of OPTN [128]
DA2	-	Non-proteolytic	Autoubiquitination of DA2 stabilizes itself and modulates its E3 ligase activity [129]
**K63-linked chains**			
EGFR	-	Endocytosis and protein trafficking	Internalization of EGFR upon ubiquitination and sorted into MVBs [136–138]
ULK1, Beclin1	-	Autophagy	Promote autophagy [148,149]
RagA	E3: RNF152	mTOR signaling	Negatively regulate mTOR signaling [151]
BNIP1	E3: RNF185	Cellular localization, Autophagy	Translocation from ER to mitochondria and promotes autophagy [152,153]
XRCC4	SCF^FBXW7^ E3 ligase	DNA repair	Facilitation of non-homologous end joining (NHEJ) repair [154]
NS2	-	Viral infection	Ubiquitination of hepatitis C virus NS 2 protein enhances the envelopment of virus and promotes its infection [155]
Dvl2	E3: WWP2	Wnt signaling	Causes phase separation into condensates, and induces Wnt signaling [158]
EZH2	-	Proteasomal degradation	Degradative during biliary fibrosis [162]
TXNIP1	-	Proteasomal degradation	Degradative [163]
**M1-linked chains**			
NEMO	E3: LUBAC	NF-κB signaling	Promotes NF-κB signaling [171]
OPTN	-	NF-κB signaling	NF-κB signaling and apoptosis [173]
**Mixed-linkage chains**			
Beclin1 (K6/K27 linked chains)	E3: NEDD4	Autophagy	Addition of K6/K27-linked ubiquitin chains on Beclin1 presents the addition of K48-linked degradation [178]
DJ-1, α-synuclein (K6/K27/K29 linked chains)	E3: TRAF6	Promotes aggregation	Lewy bodies in Parkinson’s disease [179]
BRAF35 (K6/K27/K29 linked chains)	E3: MID1	Proteasomal degradation	Degradative [180]
Enolase (M1/K63)	-	-	Ubiquitination on food vacuole associated enolase in plasmodium [181]
MHC I (K11/K63)	E3: MIR2 UBD: Epsin1	Degradative	Serves as an internalization signal [182]
Outer mitochondrial membrane proteins (K6/11/48/63 linked chains)	E3: Parkin	Mitophagy	Degradative and promotes mitophagy [47,183]
**Branched-linkage chains**			
PHF-Tau (K48/K6/K11 linked chains)	-	Tau aggregates	K6/K11 branching on K48-linked ubiquitin chains to prevent proteasomal degradation [188]
(K48/K29 linked chains)	E3: Ufd4p E4: Ufd2p, E3, Ufd4, Ubr1	ERAD and stress response, Targeting to proteasome	K48 linkages on pre-existing K29-linked ubiquitin chains to prime the substrate for degradation [189–191]
p53 (K11/K29/K63 linked chains)	-	DNA damage	Stabilizes p53 in response to DNA damage [195]

- While lysine ubiquitination has been extensively characterized, studies depicting ubiquitination on non-lysine residues such as cysteine, serine, and threonine [257–262] suggest a broader and underexplored regulatory layer in ubiquitin signaling. Future studies will be required to define its abundance, enzymatic regulators, functional consequences, and to develop analytical approaches capable of reliably detecting these modifications.
- Beyond protein substrates, ubiquitin can also be conjugated to non-amino-acid moieties. Recent studies indicate that ubiquitin can form ester-linked conjugates on ADP-ribose modifications [263], and other biomolecules such as bacterial lipopolysaccharides, lipids, sugars, nucleotides, and small amine-containing metabolites [264–272]. These findings expand the conceptual scope of ubiquitin signaling to include non-proteinaceous targets. Whether such unconventional ubiquitination events occur broadly in cells, and how these atypical conjugates are generated, regulated, and construed, remain important open questions for future research.
- Emerging evidence for cross-talk between ubiquitination and ADP-ribosylation as exemplified by MARUbylation, in which DELTEX RING E3 ligases attach ubiquitin to the hydroxyl group of ADP-ribose on ADP-ribosylated substrates. The resulting ADPr–Ub conjugates are subsequently recognized by RING-UIM E3 ligases, which extend K11-linked ubiquitin chains, illustrating a hierarchical mechanism that integrates ADP-ribosylation into ubiquitin signaling networks [263,273–275]. These findings highlight an emerging layer of PTM interplay and underscore the need to understand how such hybrid modifications are functionally interpreted within ubiquitin-dependent pathways.

Ubiquitin continues to surprise us with unexpected mechanisms and regulatory roles and reinforces its central importance in shaping protein fate and function across countless cellular processes. Moving forward, a deeper understanding of how distinct ubiquitin chains are synthesized, attached to substrates, and selectively recognized *in vivo* will be key to decipher how specificity is achieved in this system. It is equally imperative to uncover how ubiquitin-binding proteins translate chain recognition into defined cellular responses. Together, these insights will not only enrich our mechanistic view of ubiquitin signaling but also pave the way for targeted therapeutic strategies in diseases driven by its dysregulation.

## Open Access

This article has been published open access under our Subscribe to Open programme, made possible through the support of our subscribing institutions, learn more here: https://portlandpress.com/pages/open_access_options_and_prices#conditional

## Abbreviations

APC/C: anaphase-promoting complex/cyclosome
DUB: de-ubiquitinase
EGFR: epidermal growth factor receptor
ERAD: ER-associated degradation
HECT: homologous to E6-AP carboxyl terminus
LUBAC: linear ubiquitin chain assembly complex
NEMO: NF-κB Essential Modulator
NZF: Npl4 zinc finger
OTU: ovarian tumor
PTM: post-translational modifications
RBR: RING-between-RING
RING: really interesting new gene
TUBE: tandem ubiquitin-binding entities
UBA: ubiquitin-associated domain
UBAN: ubiquitin binding in ABIN and NEMO
UBD: ubiquitin-binding domain
UIM: ubiquitin-interacting motif
USP: ubiquitin-specific protease
Ub: ubiquitin
Ub-AQUA: ubiquitin absolute quantification
UbiCRest: Ub chain restriction analysis
VCP: valosin-containing protein
